# Persistence of Symptoms After Discharge of Patients Hospitalized Due to COVID-19

**DOI:** 10.3389/fmed.2021.761314

**Published:** 2021-11-22

**Authors:** Lili Wu, Yongxin Wu, Haiyan Xiong, Biqi Mei, Tianhui You

**Affiliations:** ^1^Nursing Department, People's Hospital of Longhua, Shenzhen, China; ^2^School of Nursing, Guangdong Pharmaceutical University, Guangzhou, China

**Keywords:** COVID-19, patient discharge, long-term effect, SARS-CoV-2, review

## Abstract

Many patients who had coronavirus disease 2019 (COVID-19) had at least one symptom that persisted after recovery from the acute phase. Our purpose was to review the empirical evidence on symptom prevalence, complications, and management of patients with long COVID. We systematically reviewed the literature on the clinical manifestations of long COVID-19, defined by the persistence of symptoms beyond the acute phase of infection. Bibliographic searches in PubMed and Google Scholar were conducted to retrieve relevant studies on confirmed patients with long COVID that were published prior to August 30, 2021. The most common persistent symptoms were fatigue, cough, dyspnea, chest pains, chest tightness, joint pain, muscle pain, loss of taste or smell, hair loss, sleep difficulties, anxiety, and depression. Some of the less common persistent symptoms were skin rash, decreased appetite, sweating, inability to concentrate, and memory lapses. In addition to these general symptoms, some patients experienced dysfunctions of specific organs, mainly the lungs, heart, kidneys, and nervous system. A comprehensive understanding of the persistent clinical manifestations of COVID-19 can improve and facilitate patient management and referrals. Prompt rehabilitative care and targeted interventions of these patients may improve their recovery from physical, immune, and mental health symptoms.

## Introduction

Coronavirus disease 2019 (COVID-19) is an acute respiratory infectious disease caused by a novel coronavirus, severe acute respiratory syndrome coronavirus 2 (SARS-CoV-2), that was first reported in Wuhan at the end of December 2019 (1, 2). Patients with COVID-19 may present with a wide range of clinical manifestations, including asymptomatic infection, fatigue, dyspnea, myalgia, mild upper respiratory illness, life-threatening severe viral pneumonia, or death. As of 6 August 2021, there were more than 200-million confirmed cases of COVID-19 and more than 4.26 million deaths worldwide (3). After some patients were discharged, doctors have observed persistent symptoms and unexpected substantial organ dysfunction, similar to that previously reported in some patients who had SARS (3). The criteria for discharge of patients who were hospitalized with COVID-19 vary among countries, and also according to specific epidemiological conditions and other factors, but typically include significant resolution of clinical symptoms, several negative RT-PCR test results, and serological test results indicative of specific IgG. A significant number of COVID-19 patients continue to experience symptoms and complications for several months after recovery from acute disease. This clinical manifestation has been termed “long COVID,” “chronic COVID-19,” or “post-acute COVID-19 syndrome” in the scientific literature because there is no consensus on the terminology (4–6). The current National Institute for Health and Care Excellence guideline from the United Kingdom suggests that “long COVID” includes both ongoing symptomatic COVID-19 (signs and symptoms of COVID-19 lasting 4 to 12 weeks) and “post-COVID-19 syndrome” (signs and symptoms of COVID-19 continuing beyond 12 weeks without an alternative diagnosis) (7).

However, there is incomplete knowledge about these patients after hospital discharge, because most researchers have focused on hospitalized patients who were seriously ill or elderly. In fact, most patients hospitalized with COVID-19 are classified as “recovered” following discharge, and data on these patients are limited, with only short-term follow-up data typically available. There is an urgent need to systematically track the clinical manifestations in these “recovered” patients after hospital discharge. Therefore, this review aims to summarize primary research evidence on symptom prevalence, complications, and management of long COVID so that clinicians have the most up-to-date information regarding clinical practices to be used for these patients.

## Methods

This systematic review and search were conducted based on the Preferred Reporting Items for Systematic Reviews and Meta-Analyses (PRISMA) guidelines (8). Bibliographic searches in PubMed and Google Scholar were performed to identify eligible records up to August 10, 2021. There were no restrictions regarding language, type of data, or patient age, sex, or ethnicity. The search terms included “SARS-CoV-2,” “COVID-19,” “Coronavirus 19,” “long-term,” “Persistent symptoms,” and “post discharge.” We also performed additional searches of the lists of references of the included studies. All included studies were original human studies of COVID-19 patients with ongoing symptomatic COVID-19 or post-COVID syndrome. Studies were excluded if the COVID-19 patients did not recover or were not discharged; if they did not examine adults; or if they were commentaries, narrative or systematic reviews, pre-clinical studies, or meta-analyses. All records were managed using EndNote version X7.1.1.

Two authors (Lili Wu and Yongxin Wu) first screened the articles independently by the Title and Abstract, and then reviewed the full text of the remaining articles using the eligibility criteria. The risk of bias among the studies was assessed independently by three authors (Tianhui You, Biqi Mei, and Haiyan Xiong) using an appropriate checklist. In addition, two experienced researchers independently assessed the quality of each included study based on the key features of study design, which included the rigor of the statistical model, measurement of key variables, and underlying data quality. Data were double-extracted and any discrepancies were resolved by the third author before making the final decision. Information on each included study included name of the first author, country where the study was performed, sample size, patient age, follow-up days, and symptoms or complications in discharged COVID-19 patients.

## Results

**Figure 1:**
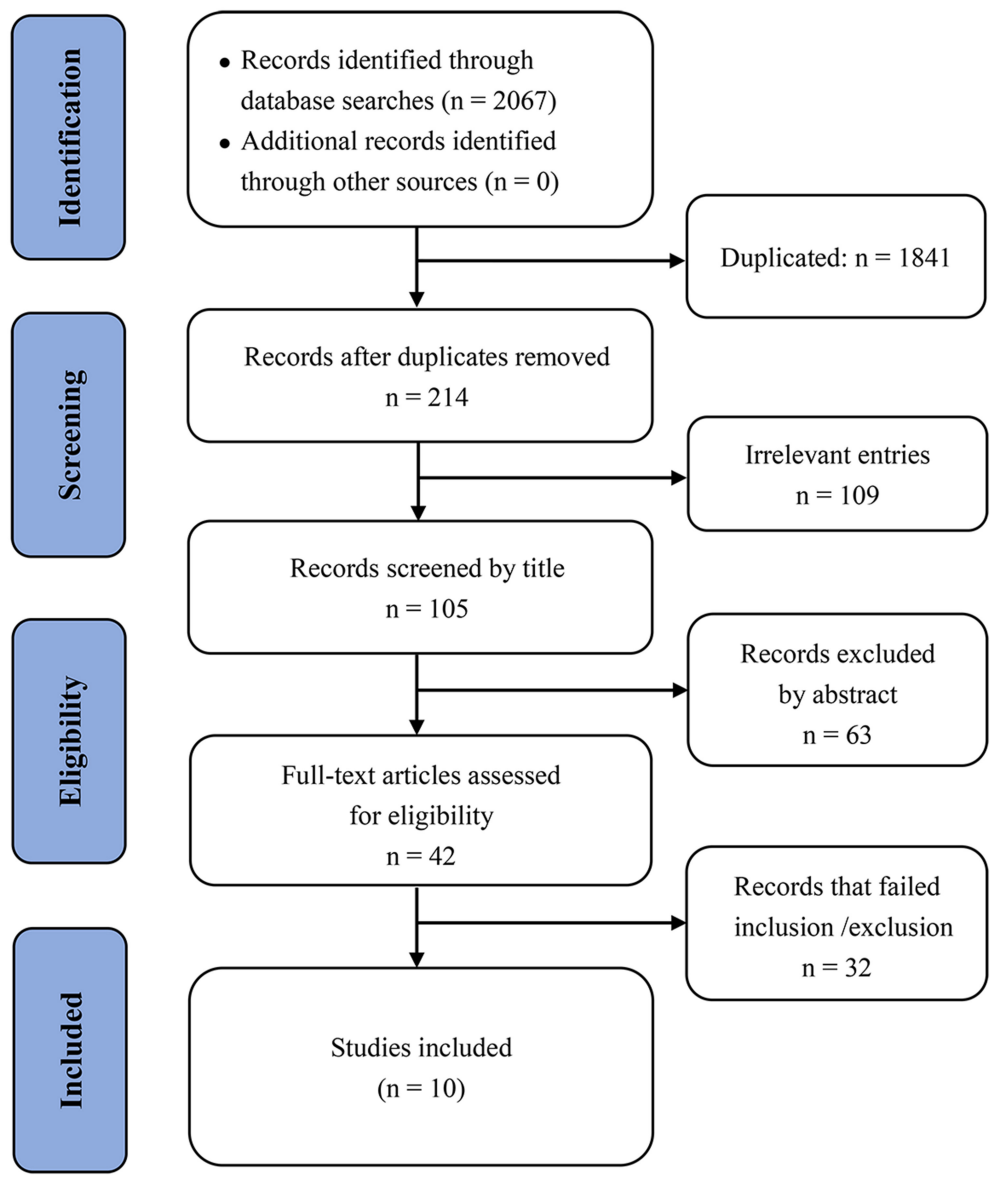
Prisma flow diagram of article selection.

We identified 2,067 records through the bibliographic database searches. After electronically removing 1,841 duplicated articles and manually excluding 172 obviously irrelevant studies (based on title and abstract), we assessed 42 publications in detail to determine eligibility, and ultimately included 10 studies for the evidence synthesis (Figure 1).

The epidemiologic and clinical manifestations of the patients described in the studies are shown in Table 1. The eligible studies were from 6 countries, and there were relatively high numbers of patients from China (1,733 patients) and Italy (402 patients). A wide range of physical and psychological symptoms were observed in patients with long COVID after discharge, including cough, sputum production, dyspnea, chest tightness, chest pain, myalgia, fever, dizziness, headaches, arthralgia, diarrhea or vomiting, sore throat or difficulties in swallowing, ageusia, anosmia, hair loss, palpitations, fatigue or muscle weakness, anxiety or depression, and sleep difficulties (Table 2).

**Table 1:** Characteristics of included studies.

**Authors**	**Location**	**Design**	**Age, years**	**Sample, *n***	**Gender, (Male/Female)**	**Follow-up, days**	**Risk of bias^a^**	**Quality assessment**
Huang et al. (9)	Wuhan, China	Cohort study	57 (47, 65)	1733	897/836	153 (146, 160)	Low	Good
Halpin et al. (10)	Leeds, UK	Cohort study	66.66 (20, 93)	100	54/46	48 (29, 71)	Low	Good
Mazza et al. (11)	Milan, Italy	Cohort study	57.8 (18, 87)	402	264/138	31.29 (15.7)	Low	Good
Carfi et al. (12)	Rome, Italy	Case series	56.5 (14.6)	143	90/53	36.1 (12.9)	Low	Fair
Xiong et al. (13)	Wuhan, China	Cohort study	52 (22, 79)	538	245/293	97 (91, 116)	Low	Good
Mandal et al. (14)	London, UK	Case series	59.9 (16.1)	384	238/146	54 (47, 49)	Moderate	Fair
Garrigues et al. (15)	Clichy, France	Case series	63.2 (15.7)	120	75/45	110.9 (11.1)	Low	Good
Zhao et al. (16)	Henan, China	Cohort study	47.74 (15.49)	55	32/23	3^*^	Low	Good
Taboada et al. (17)	Northwestern, Spain	Case series	65.5 (10.4)	91	59/32	6^*^	Moderate	Fair
Liang et al. (18)	Wuhan, China	Cohort study	41.3 (24, 76)	76	21/55	3^*^	Low	Good

a *Risk of bias of each case is assessed by the Joanna Briggs Institute critical appraisal tools for case reports and prevalence studies*;

* *months*.

*Patient age and follow-up time are given as median (IQR) or mean (SD), and all other data as n (%)*.

**Table 2:** Signsand symptoms of long COVID in COVID-19 patients after hospital discharge.

**Authors**	**Fatigue or muscle weakness**	**Sleep difficulties**	**Cough**	**Dyspnea**	**Chest pain**	**Myalgia**	**Anxiety or depression**	**Fever**	**Dizziness**	**Arthralgia**	**Diarrhea or vomiting**	**Ageusia**	**Anosmia**	**Chest tightness**	**Hairloss**	**Sputum**	**Headaches**	**Palpitations**	**Sore throat or difficulties to swallow**
Huang et al. (9)	1,038/1,655 (63)	437/1,655 (26)	NA	419/1,615 (26)	75/1,655 (5)	39/1,655 (2)	367/1,617 (23)	2 (1^**^)	101/1,655 (6)	154/1,655 (9)	80/1,655 (5)	120/1,655 (7)	176/1,655 (11)	NA	359/1,655 (22)	NA	33/1,655 (2)	154/1,655 (9)	69/1,655 (4)
Halpin et al. (10)	64 (64)	NA	NA	50 (50)	NA	NA	23 (23)	NA	NA	19 (19)	NA	NA	NA	NA	NA	NA	NA	NA	25 (25)
Mazza et al. (11)	NA	161 (40)	NA	NA	NA	NA	126 (31)	NA	NA	NA	NA	NA	NA	NA	NA	NA	NA	NA	NA
Carfi et al. (12)	76 (53.1)	NA	23 (16.1)	62 (43.4)	31 (21.7)	9 (6.3)	NA	0 (0)	9 (6.3)	39 (27.3)	5 (3.5)	15 (10.5)	21 (14.7)	NA	NA	12 (8.4)	13 (9.1)	NA	10 (7)
Xiong et al. (13)	152 (28.3)	95 (17.7)	38 (7.1)	150 (25.0)	66 (12.3)	24 (4.5)	35 (6.5)	NA	14 (2.6)	41 (7.6)	NA	NA	NA	76 (14.1)	154 (28.6)	16 (3)	NA	NA	17 (3.2)
Mandal et al. (14)	69	NA	381 (34)	204 (53)	NA	NA	56 (14.6)	NA	NA	NA	NA	NA	NA	NA	NA	NA	NA	NA	NA
Garrigues et al. (15)	66 (55)	37 (30.8)	20 (16.7)	50 (41.7)	13 (10.8)	NA	NA	NA	NA	NA	NA	13 (10.8)	16 (13.3)	NA	24 (20)	NA	NA	NA	NA
Zhao et al. (16)	9 (16.4)	NA	1 (1.8)	8 (14.5)	NA	NA	NA	NA	NA	NA	17 (30.9)	2 (3.6)	NA	NA	NA	NA	10 (18.7)	NA	NA
Taboada et al. (17)	34 (37.4)	28 (30.8)	13 (14.4)	76 (85.7)	8 (8.8)	34 (37.4)	6 (6.6)	NA	NA	26 (28.6)	NA	NA	10 (11.0)	NA	NA	NA	NA	NA	NA
Liang et al. (18)	45 (59)	NA	45 (59)	46 (61)	NA	NA	NA	15 (20)	NA	NA	20 (26)	NA	NA	47 (62)	NA	33 (43)	NA	47 (62)	NA

*Data are shown as n (%). NA, not applicable*.

** *Please use actual values instead. The text “Number less than 1” is imprecise*.

Based on the quality assessment criteria, the included studies had mixed quality (Table 1). Seven studies were assessed as good quality because they were well-designed and used robust statistical models. The remaining three studies had fair quality because they reported unreliable results from subjective questionnaires (17), lacked matched controls (12, 14, 17), or provided limited descriptions of methods (14, 17).

### General Symptoms

COVID-19 patients hospitalized with more severe disease are more likely to suffer from long-term symptoms than not requiring hospitalization. For example, the results of a follow-up study in Wuhan indicated that 76% of COVID-19 patients who were hospitalized had at least one common symptom after 6 months, and this percentage was greater in women (9). The most common symptoms were fatigue or muscle weakness, difficulty breathing, difficulty sleeping, and hair loss. The results of another study of COVID-19 survivors in Wuhan indicated that physical decline, fatigue, post-activity dyspnea, and hair loss were more common in women than men (13). However, the results of a single-center study of post-discharge persistent symptoms indicated there were no statistically significant differences in common symptoms between general ward patients and intensive care unit (ICU) patients who had COVID-19 (15). About 87.4% of Italian patients who recovered from COVID-19 experienced at least one persistent symptom, and fatigue and breathing difficulties were the most common (12). In addition, researchers from the United Kingdom showed that fatigue was the most common symptom at 7 weeks (72%), and that 53% of patients reported a clinically significant decrease in quality of life (10). The results of a study from South Korea indicated that most COVID-19 survivors experienced several mild impairments during the 3 months after hospital discharge (18).

The present summary of recent publications on the long-term health consequences of COVID-19 after hospital discharge indicates that fatigue, dyspnea, chest tightness, cough, joint pain, muscle pain, loss of taste/smell, hair loss, sleep difficulties, and anxiety or depression were the most common symptoms (Table 1). These symptoms are similar to the long-term consequences previously reported by patients who had SARS (19). In addition to these general symptoms, other studies have reported symptoms in specific organs or organ systems, mainly the lungs, heart, kidneys, and nervous system.

### Pulmonary Symptoms

The lungs are the organs most affected in patients with COVID-19 (20, 21). Similarly, earlier studies found that patients who recovered from coronavirus pneumonia due to SARS-CoV-1 or Middle East Respiratory Syndrome-related coronavirus (MERS-CoV) experienced impaired lung function that lasted for months or even years (19, 22, 23).

A brief report of 18 COVID-19 patients from China after hospital discharge showed that 15 of them (83.3%) had evidence of residual abnormalities based on chest computed tomography (CT), and the most common manifestations were ground glass opacities (GGO) and pulmonary fibrosis (24). In another study, Chinese researchers examined 59 discharged patients and reported that 39% of them developed fibrosis, and that COVID-19 appeared to increase the risk of early pulmonary fibrosis after discharge (25). Their analysis of thin-slice CT results indicated that elderly patients with severe diseases during the treatment period were more likely to have lung fibrosis (21, 24–26). This results of this study also suggested that interstitial thickening, irregular interfaces, thick reticular structures, and solid bands may be predictors of pulmonary fibrosis during the course of disease. There is also evidence that interfacial irregularities and parenchymal bands predict the early formation of pulmonary fibrosis (21).

The results of a study of 55 patients with COVID-19 in China indicated that 39 of them (71%) had varying degrees of radiographic abnormalities at 3 months after discharge, including pure ground-glass opacity (7 of 55, 7.27%), interstitial thickening (15 of 55, 27.27%), and crazy paving (3 of 55, 5.45%) (16). Even though most patients were free of respiratory symptoms at follow-up, lung function abnormalities were also present in 14 patients (25.45%) (16). Similarly, Huang et al. (9) reported that 57 COVID-19 survivors had abnormal pulmonary function test results at 30 days after discharge that could have serious adverse cardiopulmonary consequences if accompanied by cardiovascular comorbidities and continued decline in pulmonary function. Liu et al. (27) reported that lung damage in typical COVID-19 patients was reversible, and their CT scans of 51 COVID-19 patients at 4 weeks after discharge showed complete resolution of lung damage in 64.7% of these patients. However, long-term CT follow-up studies are required to determine whether some patients with COVID-19 develop irreversible fibrosis.

### Cardiovascular Symptoms

The *Clinical Bulletin of the American College of Cardiology* concluded that myocardial injury was a complication related to COVID-19 (28). Researchers in Germany recently assessed the long-term consequences of 100 patients who recovered from COVID-19. At a median time of 71 days after initial diagnosis, cardiovascular magnetic resonance imaging (CMRI) showed heart involvement in 78 cases (78%) and continuous myocardial inflammation in 60 cases (60%) that were independent of preexisting complications. There was also no relationship of these conditions with the duration or severity of acute COVID-19, nor with the time since initial diagnosis (29).

In addition, the results of a recent study of 538 COVID-19 patients undergoing rehabilitation in Wuhan indicated that 70 patients (13%) had cardiovascular symptoms after 3 months; in addition, 60 patients (11%) had significantly greater resting heart rates (more than 20 beats per minute greater than before COVID-19 diagnosis), and 45 of these 60 patients experienced increased heart rates in the hospital that remained after discharge. Another 26 patients (4.8%) said that they occasionally had palpitations and 17 patients (3.2%) had new-onset of hypertension (13). However, further investigations are needed to confirm the presence of long-term cardiovascular consequences after COVID-19.

### Kidney Symptoms

Acute kidney injury (AKI) is common during COVID-19 hospitalization, but there is limited evidence that persistent renal damage is among the long-term sequelae. The results of a 2017 indicated that persistent renal impairment may occur after the onset of AKI and may progress to end-stage renal disease, necessitating dialysis (28). The results of a study of renal complications in COVID-19 patients from Hong Kong indicated a low overall rate of AKI, but that patients with nosocomial AKI had prolonged hospital stays and renal function impairment that persisted up to 6 months after discharge (30). Chaolin Huang and colleagues performed a cohort study of 1,733 adults with COVID-19 who were discharged from Jinyintan Hospital (Wuhan, China). A total of 35% (479/1,378) had low estimated glomerular filtration rates (eGFRs) at different times after follow-up, and 13% (107/822) without AKI and normal renal function during the acute phase of COVID-19 had decreased eGFRs (<90 mL/min/1.73 m^2^) at different times during follow-up (9). More evidence is needed to determine whether kidney damage after discharge is a long-term consequence of COVID-19, and the possible pathogenic mechanism.

### Neurological Symptoms

Researchers who performed a 3 month follow-up study of COVID-19 patients reported impaired microstructural and functional integrity of the brain based on magnetic resonance imaging (MRI), and that 55% of the patients had persistent neurological symptoms (31). By far, the most common long-term neurological symptoms after COVID-19 were headache, insomnia, and loss of taste or smell, followed by excessive sweating, dizziness, tinnitus, and several other conditions (9). Researchers who performed another follow-up study of 324 COVID-19 patients after discharge reported that 12.07% of them had headaches or insomnia (27), and the results of another study of 120 COVID-19 patients at 3 months after discharge indicated that up to 30.8% of them had sleep disturbances (15), although only 17.7% of patients had sleep disturbances in a different follow-up study (13). The results of a study in Wuhan indicated that 23.6% of COVID-19 survivors had excessive sweating (13). In addition, the results of a medium-term follow-up study of 337 patients indicated that 10.5% of them had other neurological symptoms, including dizziness, wakefulness, and tinnitus (32).

Neurological injury is often accompanied by mental disorders, such as depression, anxiety, sleep disorders, migraine, etc. These mental disorders can have detrimental impacts on the outcome of neurological injury, and also decrease quality-of-life (33). The results of an increasing number of studies indicated that COVID-19 patients experienced different types of psychological distress after discharge, mainly anxiety/depression and post-traumatic stress disorder (PTSD) (34). Researchers in Spain performed a follow-up study of 91 survivors of critical COVID-19 found that 46% of them had moderate levels of anxiety/depression after more than 6 months (17). The results of another follow-up study of 122 patients after discharge indicated that the most common clinical manifestations were psychosocial symptoms (22.7%) (13). The results of a survey of post-discharge psychological distress indicated that COVID-19 patients who received intensive care treatments were twice as likely to have adverse psychological effects as those who did not (46.9% vs. 23.5%) (10). These results suggest that COVID-19 patients with more serious diseases have an increased risk of psychological distress. Italian researchers examined 113 COVID-19 patients after discharge and found that 33% of them had depression (35).

In addition to anxiety and depression, clinicians must also be cognizant of PTSD in COVID-19 patients after discharge. The symptoms of PTSD include fear of death, intrusive thoughts, insomnia, feeling alienated or isolated from others, and inability to concentrate. The results of a recent study in China indicated that up to 96.2% of clinically stable COVID-19 patients had clinically significant symptoms of PTSD (36). However, the results of the afore-mentioned Italian study, which followed the psychological status of 238 COVID-19 patients for 4 months after discharge, indicated that only 17.2% of them had symptoms of PTSD (35). Patients infected with COVID-19 may experience various stressors or major traumatic events, such as physical and social isolation or the death of family members (36, 37). The perceived stigma of COVID-19 and a previous history of psychiatric treatment may influence the severity of PTSD. For example, 40% of COVID-19 survivors said they were concerned about infecting others and being discriminated against by their social contacts. Patients who recovered from COVID-19 expected support from neighbors and family members and regular hospital check-ups after hospital discharge (34, 38).

The results of an Italian study of patients with COVID-19 indicated that many of them had mental health disorders at 1 month after discharge, in that 28% had PTSD, 31% had depression, 42% had anxiety, 20% had obsessive-compulsive disorder, and 40% had insomnia. In addition, women and patients with histories of previous mental distress were more likely to experience psychological problems, and young patients were more likely to experience sleep disorders and depression (11). Previous studies of SARS and MERS patients showed that the prevalences of depression, anxiety, and PTSD remained high after 39 months (39). We therefore anticipate that patients hospitalized with COVID-19 will continue to experience significant and long-term mental health symptoms after discharge.

## Discussion

There is currently no consistent definition for the wide spectrum of conditions that may be experienced by patients after resolution of acute COVID-19. The term “long COVID” is used to describe people who recovered from acute disease but still report long-term effects from the infection or common clinical symptoms that last much longer than expected (14). For some of these patients, the return to their previous state of health is slow and uncertain. This includes patients who recovered from severe acute illness and those who had mild to moderate disease (12). The SARS-CoV-2 virus can infect the lungs, heart, gastrointestinal mucosa, liver, and kidney, vascular endothelium, T lymphocytes, macrophages, and neurons (40). Thus, the long-term complications experienced by these patients may be the result of direct viral invasion of these tissues, in which the virus binds to the angiotensin-converting enzyme 2 (ACE2) receptor, and then activates a cytokine storm, immune system damage, or a combination of these (41, 42), culminating in extensive tissue damage (43). Thus, there may be specific sequelae in each affected organ. The World Health Organization (WHO) recommended a maximum recovery time of 2 weeks for patients with mild disease and 6 weeks for those with severe disease (44). However, our literature search showed that many patients who recovered from acute disease continued to suffer from long-term sequelae. Because of the very large number of people who had COVID-19, this is a significant long-term burden on the health care systems of many countries.

### General Symptoms

The current evidence suggests that the most common long-term clinical manifestation after discharge of adults with COVID-19 is fatigue, which is not surprising given that post-infection fatigue also occurred in patients who were infected by numerous other viruses (45, 46). Recent studies found an association between single nucleotide polymorphisms (SNPs) in certain cytokine genes of patients with post-infection complications, such as fatigue, pain, neurocognitive impairment, and mood disorders, and that the “cytokine storm” experienced by these patients during the acute phase of disease may persist and lead to long-term fatigue (47).

### Pulmonary Symptoms

At discharge, COVID-19 patients may still have breathing difficulties. Some researchers suggested that chest CT imaging should be used to quantify the extent of pulmonary perfusion/ventilation for diagnosis, and that changes in pulmonary microcirculation should be monitored over time to identify possible complications and the need for pharmacological interventions (48). Some patients have chronic pulmonary fibrosis after discharge, and this can lead to impaired lung function. Evidence from the previous SARS and MERS pandemics suggests that some patients may even experience lung damage up to 15 years after the onset of infection (49).

### Cardiovascular Symptoms

Pericarditis and myocarditis may be present long after resolution of a SARS-CoV-2 infection. This is important to consider for all infected individuals, not just those who were hospitalized, and may indicate that cardiac sequelae are associated with delayed adaptive and inborn immune responses that are altered (50). Adults hospitalized with COVID-19 may still be at risk for coronary artery disease or ventricular arrhythmia due to myocardial injury, even after significant recovery of cardiac function (51).

### Kidney Symptoms

The renal damage that occurs during COVID-19 varies greatly among patients. Recent studies found that the progression of acute kidney injury (AKI) in patients with severe COVID-19 leads to significantly worse prognosis. The pathological changes in the kidneys may be due to cytopathic effects caused by local replication of the virus during the acute phase of disease. Alternatively, these changes may occur indirectly due to a systemic immune response or excessive hemagglutination (immunothrombosis) (52).

### Neurological Symptoms

SARS-CoV-2 may cause anosmia through direct attack of the olfactory nerve, or may penetrate brain tissue through viremia and cause a wide range of other neurological symptoms, such as tinnitus and headache (53). In particular, the virus may alter the synaptic transmission of peripheral nerves and lead to abnormal permeability of blood-brain barrier channel proteins due to its binding to the ACE2 receptor, and thus enter the brain using a “Trojan horse” strategy (54, 55). The SARS-CoV-2-mediated immunopathology and colonization of the intestinal system and central nervous system, as well as the systemic inflammatory responses during the acute phase of COVID-19, could also lead to neurodegenerative diseases and chronic autoimmune diseases (56).

Regarding mental health, a coronavirus infection may lead to psychiatric problems by several possible mechanisms. For example, the virus may directly infect the brain and alter its function. Alternatively, COVID-19 may promote cerebrovascular disease and induce hypercoagulation, leading to physical damage from hypoxia and altered immune responses. These patients require prompt medical interventions and experience social isolation during treatment. Moreover, facing a serious and potentially fatal disease can have a significant psychological impact. Many other patients with less severe disease experience fear due to the stigma of infection (39). Viral infections are common, and some viruses can infect the central nervous system, causing neuropsychiatric syndromes that affect the cognitive, emotional, behavioral, and perceptual domains (57–59). COVID-19 has widespread effects on the mental health of patients that should be considered after patient discharge.

### Limitations

Our review has several limitations. Two authors independently searched for eligible studies, and the third author repeated the search process multiple times, but we cannot guarantee that all of the most recent latest publications are included. Moreover, most of the included studies enrolled relatively small numbers of patients with confirmed SARS-CoV-2 infections. Given the fast and constantly evolving nature of the pandemic, some of the information in these publications should be considered somewhat provisional. In addition, some of the included studies were case series and cross-sectional studies, and these results do not allow inferences about causation, but only relationships. We therefore cannot ensure that all reported symptoms were actually associated with long COVID, and cannot rule out the possibility of secondary causes. Therefore, only narrative descriptions of study findings were provided in this review.

## Conclusion

Because COVID-19 is a recent disease, there has not been enough time to examine the long-term effects on patients after they recover from the acute phase of disease. Over time, as more long-term data become available, we expect that our understanding of the long COVID will improve. Nonetheless, it is clear that many COVID-19 survivors experience impaired physical and mental function after hospital discharge. Our review has several clinical implications. In particular, we suggest the use of early rehabilitation interventions during the post-hospitalization stage, and active rehabilitation training and interventions for patients with respiratory dysfunction, physical symptoms, and mental disorders, to restore their physical, immune, and mental health as much as possible. It is imperative that a multidisciplinary approach be used to care for this large and vulnerable population. Comprehensive long-term studies of the effects of COVID-19 on multiple organ systems are needed to safeguard the physical and mental health of the tens of millions of patients who survived COVID-19.

## Author Contributions

LW: conceptualization, data curation, formal analysis, validation, and writing—original draft. YW: Data curation, formal analysis, validation, and writing—review and editing. HX: conceptualization, formal analysis, validation, and writing—original draft. BM: investigation, supervision, validation, and writing—review and editing. TY: conceptualization, project administration, supervision, and writing—review and editing. All authors contributed to the article and approved the submitted version.

## Conflict of Interest

The authors declare that the research was conducted in the absence of any commercial or financial relationships that could be construed as a potential conflict of interest.

## Publisher's Note

All claims expressed in this article are solely those of the authors and do not necessarily represent those of their affiliated organizations, or those of the publisher, the editors and the reviewers. Any product that may be evaluated in this article, or claim that may be made by its manufacturer, is not guaranteed or endorsed by the publisher.

## Abbreviations

COVID-19: coronavirus disease 2019
SARS-CoV-2: severe acute respiratory syndrome coronavirus 2
SARS: severe acute respiratory syndrome
ICU: intensive care unit
MERS-CoV: Middle East Respiratory Syndrome-related coronavirus
CT: chest computed tomography
GGO: ground glass opacities
MRI: magnetic resonance imaging
AKI: acute kidney injury
eGFRs: estimated glomerular filtration rates
PTSD: post-traumatic stress disorder
ACE2: angiotensin-converting enzyme 2
WHO: World Health Organization
SNPs: single nucleotide polymorphisms.
